# Alterations in the expression and activity of extracellular matrix components in HPV-associated infections and diseases

**DOI:** 10.6061/clinics/2018/e551s

**Published:** 2018-08-28

**Authors:** Suellen Herbster, Andressa Paladino, Sumara de Freitas, Enrique Boccardo

**Affiliations:** Laboratory of Oncovirology, Department of Microbiology, Institute of Biomedical Sciences, University of São Paulo, São Paulo, Brazil

**Keywords:** Human papillomavirus, Extracellular matrix, Matrix Metalloproteinases, TIMPs, RECK

## Abstract

Infection with human papillomaviruses is associated with a series of benign and malignant hyperproliferative diseases that impose a heavy burden on human populations. A subgroup of mucosal human papillomavirus types are associated with the majority of cervical cancers and a relevant fraction of vulvar, vaginal, anal, penile and head and neck carcinomas. Human papillomaviruses mediate cell transformation by the expression of two pleiotropic oncoproteins that alter major cellular regulatory pathways. However, these viruses are not complete carcinogens, and further alterations within the infected cells and in their microenvironment are necessary for tumor establishment and progression. Alterations in components of the extracellular matrix for instance, matrix metalloproteinases and some of their regulators such as tissue inhibitors of metalloproteinases, have been consistently reported in human papillomaviruses-associated diseases. Matrix metalloproteinases function by remodeling the extracellular matrix and alterations in their expression levels and/or activity are associated with pathological processes and clinical variables including local tumor invasion, metastasis, tumor relapse and overall patient prognosis and survival. In this review we present a summarized discussion on the current data concerning the impact of human papillomavirus infection on the activity and expression of extracellular matrix components. We further comment on the possibility of targeting extracellular matrix molecules in experimental treatment protocols.

## INTRODUCTION

Human papillomaviruses (HPVs) are small (∼50 nm), nonenveloped viruses with marked tropism for the stratified epithelia of mucosa and the skin. The viral cycle is strictly dependent on the epithelial differentiation program since the availability of cellular factors expressed in different strata of the epithelium play a role on viral gene expression and genome amplification (see below). The genome of these viruses is composed of a circular molecule of double-stranded DNA of approximately 8 kbp packaged with cellular histones. The viral DNA can be divided into three functional regions. The long control region (LCR) or upstream-regulatory region (URR) is a noncoding stretch of DNA that contains the viral origin of replication and sites for binding viral and cellular factors. The combination of viral and cellular proteins that bind to this region regulates both viral genome replication and gene transcription. In addition, HPVs exhibit two coding regions. The early region (E) codifies the early genes that regulate viral DNA replication and gene expression (E1, E2, E7), viral persistence (E5, E6 and E7), immune evasion (E5, E6 and E7), inhibition of apoptosis (E6 and E7) and virion maturation and release (E4). The late region (L) contains the genes that codify the major (L1) and minor (L2) capsid proteins 1.

HPV infections are ubiquitous and are associated with a series of hyperproliferative pathologies of epithelia and mucosa as well as most cases of cervical cancer and genital warts 2,3. To date, more than 200 HPV types have been described 4. Almost 40 types exhibit particular tropism for the mucosa of the anogenital region. HPV types from this subgroup are classified as being of high or low oncogenic risk, according to their association with cervical cancer. While high-risk HPV types are associated with almost the totality of cervical cancer cases, low-risk HPV types are the cause of almost all anogenital warts and low-grade lesions with little tendency to malignant progression. The most prevalent high-risk HPVs are HPV16 and HPV18, while the most common low-risk types are HPV6 and HPV11. In addition, studies conducted during the last two decades have clearly shown that infections with specific high-risk HPV types are etiologically related with a significant proportion of vulvar, vaginal, anal, penile and head and neck carcinomas 5,6.

High-risk HPV types express two oncogenes, E6 and E7, that collaborate to immortalize normal human keratinocytes in cell culture systems and are essential to maintain the transformed phenotype *in vivo* 7. The main role of these proteins during the HPV cycle is to generate a cellular microenvironment permissive for viral replication 8. This includes the induction of DNA replication machinery, immune evasion and downregulation of apoptosis 9-11. To achieve this goal, E6 and E7 target critical cellular regulatory pathways including those mastered by p53 and pRb, respectively 18-20.

Despite the high impact of the expression of HPV proteins on cellular homeostasis, these viruses are incomplete carcinogens. Therefore, further alterations in the cell as well as in its microenvironment are necessary for tumor establishment and progression. This process includes dysregulation of the extracellular matrix (ECM). In some cases, alterations in the levels and activity of defined ECM components have been experimentally associated with the expression of specific HPV proteins, suggesting the direct involvement of the virus in the deregulation of these factors. Other studies, mainly those conducted using clinical samples, have identified alterations in the levels of ECM molecules during the progression of HPV-related diseases. However, in most situations the direct involvement of viral proteins on these alterations has not been addressed. In this review we discuss the current state of knowledge concerning the impact of HPV infection on the expression and function of components of the ECM.

### ECM alterations in HPV-associated anogenital intraepithelial neoplasias

The natural history of cervical cancer development arises from precursor lesions, called cervical intraepithelial neoplasias (CINs). CIN 1 and a subgroup of CIN 2 lesions are classified as productive lesions, in which the viral cycle is complete. On the other hand, a subgroup of CIN 2 and all CIN 3 lesions are cervical cancer precursors. The development of these lesions is mainly caused by persistent infection with oncogenic HPV types. Productive lesions present low to moderate histological alterations and spontaneously regress in the period of 1 to 2 years. In the event of high-risk HPV persistent infections, high-grade precancerous lesions (a subgroup of CIN 2 and all CIN 3) may develop within 3 to 5 years. Morphologically, CIN 3 (carcinoma *in situ* [CIS]) represents a heterogeneous disease, and it can be considered a precursor lesion to a more advanced, invasive cervical cancer 21.

**Figure 1:**
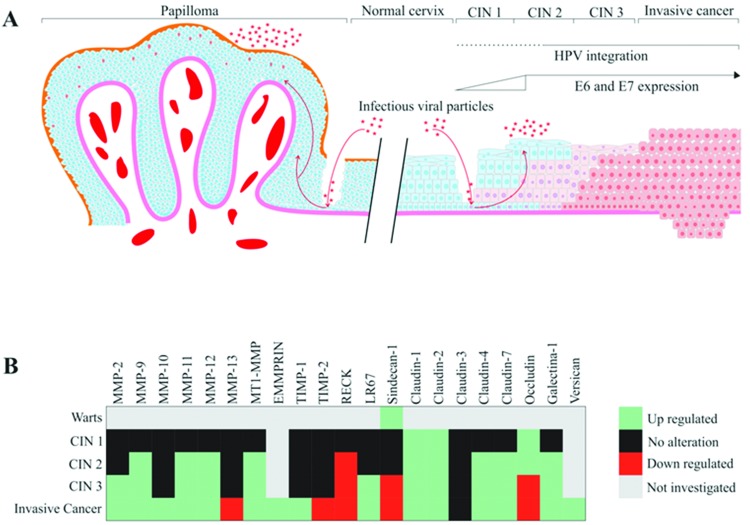
An overview of the reported ECM alteration events in HPV-associated pathologies. A) HPV infections are strongly associated with the development of skin and genital warts and cervical cancer precursor lesions. HPV gains access to the basal layer of the epithelium through microwounds and initiates the infection. HPV-associated warts are characterized by nontransforming (or benign) increased cell proliferation of the anogenital skin and mucosa due to productive viral infection. The epidermis of HPV-infected skin, mucosa and genital warts may present papillomatosis, acanthosis (increased number of cell layers), hyperkeratosis and parakeratosis. Warts frequently show elongated epithelial ridges roughly directed to the center of the wart and increased vascularization of the bordering dermis. In warts and low-grade cervical intraepithelial neoplasias (CIN 1), the HPV genome is frequently found in the episomal form. On the other hand, integration of the HPV genome and resulting higher expression of E6 and E7 oncogenes are common features of high-grade CIN (2 and 3) and invasive cervical cancer. HPV-associated lesions are characterized by the presence of keratinocytes with atypical morphology, called koilocytes, that exhibit (I) increased cell size and (II) eccentric and pyknotic nuclei bordered by a perinuclear halo. B) The heatmap presented summarizes the reported alterations in ECM molecule expression (either mRNA or protein levels) in cervical cancer precursor lesions and invasive carcinomas.

HPV infection has been associated with several alterations in tissue organization and architecture, including dysregulation of matrix metalloproteinase (MMP) expression and activity. Additionally, alterations in different molecules of the ECM can be observed in HPV-associated CINs. The main alterations in ECM components in CIN are summarized in Figure 1. For example, when MMP-2 expression was analyzed in cervical samples, the levels of this protein were undetectable in normal cervix and low-grade lesions. In contrast, high-grade lesions and invasive carcinomas showed high MMP-2 expression, suggesting that MMP-2 may constitute an early marker for cervical cancer progression. Additionally, these results suggest that upregulated MMP-2 expression may be associated with the potential risk of invasion and metastasis 22,23. MMP-2 is also called gelatinase A and has fibronectin type II repeats in its catalytic domain, which mediates collagen binding to this protease. MMP-2 major function involves collagen type IV breakdown in the basement membrane 24,25.

MMP-2 and its regulator tissue inhibitor of metalloproteinases type 2 (TIMP-2) are important determinants in the behavior of invasive tumors and in their metastatic potential. A previous study conducted with clinical samples from patients with CIN or invasive squamous cell cervical carcinoma (CC) showed that MMP-2 expression levels presented a positive correlation with CIN grade, and was further increased after progression to invasive cancer. However, TIMP-2 expression presented no alterations when normal tissues were compared to CIN 3 tissues 26. On the other hand, our group has shown that HPV16 E7 expression is associated with a decrease in TIMP-2 expression and function in primary keratinocytes 27. Zhou et al. 28 analyzed the expression of MMP-2, MMP-9, TIMP-1 and TIMP-2 in normal cervix, CIN cervix samples and cervical cancer samples using an immunohistochemistry (IHC) assay. Their results suggest that overexpression of these ECM components may play a key role in cervical cancer-associated lymph node invasion and metastasis.

In an early study, Davidson et al. 29 analyzed the expression of MMP-9 in cervical squamous cell carcinoma and CIN 2-3 samples and observed that MMP-9 mRNA and protein expression were elevated in both high-grade CIN and invasive squamous cell carcinoma when compared with normal samples. Therefore, MMP-9 was also suggested as a possible early marker of cervical lesion progression. No et al. 30 also detected a higher expression of MMP-9 in CIN 3 lesions than that in CIN 1 and 2 lesions. In addition, our group has shown that the expression of the MMP inhibitor (MMPI) reversion-inducing protein cysteine-rich protein with kazal motifs (RECK) is downregulated in high-grade CIN and cervical cancer samples when compared with low-grade CIN and control samples. In addition, previous data from our group demonstrated that transduction with HPV16 E6 and E7 oncogenes correlates with reduced expression of the RECK protein in organotypic cultures of primary keratinocytes. We also observed increased MMP-2 and MMP-9 expression and activity in this system 27. Altogether, these results suggest that upregulation of MMP-2 and MMP-9 expression and activity are associated with high-grade CIN. On the other hand, the MMPI RECK exhibits reduced levels of expression in these lesions 26-28,31. These results are summarized in Figure 1B.

Recently, Valdivia et al. 32 analyzed the expression of MMP-11 and MMP-12 in low-and high-grade squamous lesions (LSIL and HSIL, respectively) and cervical carcinomas. These authors observed that samples with positive expression for MMP-11 were also positive for MMP-12, and their expression increased according to lesion grade. In addition, they showed that MMP-11 and MMP-12 proteins accumulated mainly in the cytoplasm of transformed cells. On the other hand, these proteins were not detected in normal epithelia. These results indicate that MMP-11 and MMP-12 may be associated with the onset of cancer precursor lesions and suggest that increased expression of these proteins can be considered an early event during the development of preneoplastic cervical lesions. The MMP-11 gene, which is also called stromelysin-3 (ST-3), is located on chromosome 22q11.23 and has been identified in a breast cancer cDNA library. MMP-12, also known as metalloelastase or macrophage elastase, is found on chromosome 11q22.3 25,32-34.

Alterations in other ECM components have also been explored in the cervical tissue transformation context. Branca et al. 35 have found that 67-kDa laminin receptor (LR67) expression progressively increases with CIN grade. LR67 is associated with CIN 2 to CIN 3 progression, and it can be thought of as a marker of cellular proliferation in cervical tissue 26. These authors have also shown that the combined analysis of LR67 and vascular endothelial growth factor-C (VEGF-C) may improve high-grade CIN clinical detection 35.

Moreover, analysis of the expression of sindecan-1 and claudins (CLDNs) in CIN 1, 2, and 3 and CIS lesions demonstrated that sindecan-1 and CLDNs 1, 4 and 7 expression levels are similar among CIN 2 and 3, CIS and normal epithelium. However, CLDN3 was not detectable in the squamous epithelium 36.

HPV infection has also been linked to a relevant percentage of lesions in other epithelia of the anogenital tract including the vulva, vagina and anus. Vulvar, vaginal and anal cancer precursor lesions are also denominated as vulvar intraepithelial neoplasia (VIN), vaginal intraepithelial neoplasia (VAIN) and anal intraepithelial neoplasia (AIN), respectively 37. Similar to what is observed in cervical precursor lesions, VIN, VAIN and AIN also progress through grades of epithelial transformation. Previous studies have determined the HPV DNA prevalence in VAIN, VIN and AIN on four continents using PCR assays. HPV6 and HPV11 were the most prevalent HPV types found in VIN 1 and AIN. Moreover, HPV16 was detected in 75% of vulvar, vaginal and anal HPV-positive carcinomas. The same study also determined that HPV18 was detected in only 10% of the evaluated samples 38. In another study, HPV16 or HPV18 were found in 76% of VIN 2-3, 64% of VAIN 2-3, 81% of AIN 2-3 and 42% of vulvar carcinoma samples. Additionally, the same HPV types were observed in 58% of women under 56 years of age who were diagnosed with carcinoma of the vulva 39.

Finally, a retrospective study analyzed the expression of MMP-2, MMP-9, TIMP-1 and TIMP-2 by IHC in VIN 1, 2 and 3 samples and invasive vulvar carcinoma. The results from this study suggested that overexpression of MMP-2, MMP-9 and TIMP-2 proteins may be related with the progression of VIN to invasive carcinoma 40.

### ECM composition in HPV-associated cancers

The carcinogenic process naturally culminates in the overcoming of multiple ECM regulatory mechanisms in order to promote tissue invasion of established tumors 41. The dysregulation of ECM remodeling proteins is a common feature in the natural history of human cancers, resulting in an overall collapse in normal ECM composition and maintenance 42,43. This event is a devastating consequence of the imbalance between expression and activity of specific proteases and their natural negative regulators, which leads to an altered deposition-to-degradation ratio of several basement membrane structural molecules.

Disruption of ECM remodeling regulation through imbalanced proteolysis has a major role in loss-of-tissue homeostasis and pathological processes such as cancer. In cancer, this event can impact (I) tissue tension and (II) the release of chemotactic fragments of ECM components that influence the local microenvironment, as it favors (III) cell migration and recruitment of stromal, endothelial and immune cells to the tumor vicinity. The heterotypical association of cancer cells and other elements observed in the tumor microenvironment, such as inflammatory infiltrates, endothelial cells and tumor-associated fibroblasts, must also be explored in order to fully understand the alterations in ECM remodeling resulting from this intricate crosstalk 44.

Historically, the most investigated proteases present in this crossroad are the MMPs. The activity of specific MMP types such as MMP-2, MMP-9 and membrane type 1 (MT1)-MMP is upregulated in most human cancers. These MMPs play a central role in basement membrane breakdown and cell invasion, as well as in neoangiogenesis and metastasis 25,45. The excess of MMP activity generates topographical changes in the tumor microenvironment by proteolysis-associated modifications of the structural ECM scaffold. In fact, linearization, thickening and/or degradation of specific collagen are common events observed in epithelial tissue areas adjacent to tumor-associated blood vessels, where cancer cells invade. MMP activity also regulates cellular migration and release of ECM fragments with biological functions, such as growth factors 42.

The crucial role of specific MMPs in the process of carcinogenesis has set an objective task for researchers in the field to explore the potential of MMPs as therapeutic targets 46. However, the use of broad-spectrum, small-molecule MMPIs offers no clinical advantages due to the dose-limiting side effects 46,47. For example, some of the nonspecific MMP inhibitors also downregulate the TNF-α converting enzyme (TACE) and prevent the release of TNF-α receptor II (TNF-RII), which contributes to the side effect of musculoskeletal pain 47-49. Nevertheless, novel and more specific MMPIs and MMP cleavage-associated cytotoxic treatments are being tested in clinical trials as potential cancer treatment options 50-52.

Several authors have studied ECM alterations in both structural and remodeling molecules in invasive cervical cancer. More specifically, alterations in the expression/activity of galectins, collagens, proteoglycans, laminins, fibronectins, integrins and proteases and their regulators were observed in both cervical cancer samples and derived cell lines. Considering the current review, we aim to assemble a more complete view of the spectrum of ECM alterations in HPV-associated tumors, mainly regarding cervical cancer.

### MMPs in HPV-associated cancers

HPV16 seems to be associated with higher expression/activity of specific MMPs during cervical cancer progression 27,31,53,54. Indeed, the imbalance between the expression/activity of proteases such as MMPs and their negative regulators is a common feature in both cervical cancer samples and derived cell lines 22,27,31,54-56.

Several authors have reported the upregulation of specific MMPs in samples of cervical carcinoma when compared to control healthy tissue, including MMP-1, MMP-2, MMP-3, MMP-7, MMP-9, MMP-10, MMP-11, MMP-12, MMP-13, MMP-14 (MT1-MMP) and MMP-15 22,27,31,54–56. The main alterations in ECM components in cervical tumors are summarized in Figure 1. Kaewprag et al. 54 analyzed the ability of HPV16 and HPV18 oncoproteins to regulate the expression of MMP-1, MMP-2, MMP-7, MMP-9, MMP-10, MMP-11 and MMP-14 (MT1-MMP). These authors found a positive correlation between the expression level of MMPs and the cell invasion potential of C33A cells stably expressing different HPV16 oncoproteins. Furthermore, this study detected an upregulation of MMP-2 and MT1-MMP gene expression when C33A cells expressed both HPV16 E6 and E7 oncoproteins, which correlated with increased cell invasion. Conversely, silencing of HPV16 E6 and E7 expression by shRNA led to the downregulation of MMP-2 and MT1-MMP expression. This was paralleled by a reduction in the invasive potential of these cells. Altogether, these observations suggest the direct effects of HPV oncoproteins in the modulation of these MMP types. Moreover, elevated MMP-2 and MT1-MMP protein levels were observed in clinical samples of HPV16-positive invasive cervical tumors when compared to CIN and normal cervical tissues. The authors also observed that PEA3 and Sp1 binding sites present on MMP-2 and MT1-MMP promoters were essential for the transactivation activity mediated by HPV16 E6 and E7. MT1-MMP (or MMP-14) functions include ECM remodeling through proteolysis of collagen I, II, and III, fibrin, fibronectin, laminin-1, laminin-5, and vitronectin 25.

Even though MMP proteolytic network alterations are common during the progression of cervical cancer, only upregulation of the expression/activity of MMP-2 (gelatinase A) and/or MMP-9 (gelatinase B) seems to be indicative of a poor prognosis in cervical cancer patients 22,57,58. In fact, others have observed a correlation between MMP-2 and MMP-9 transcript levels and cervical tumor invasion potential 22,55,56,59. Our group has shown that SiHa and CaSki HPV-positive cervical cancer-derived cell lines present higher levels of MMP-2, MT1-MMP, and TIMP-2 than the C33A HPV-negative cell line 53. Moreover, we demonstrated an association between HPV16 E6 and E7 expression, upregulation of MMP-2 and MMP-9 mRNA and decreased levels of their natural negative regulators RECK and TIMP-2 mRNA in primary keratinocytes grown in organotypic cultures 27. Finally, we analyzed RECK and MMP-9 expression levels in clinical samples from patients diagnosed with cervical cancer and observed (I) downregulated RECK protein expression and (II) upregulated MMP-9 protein expression in cervical cancer when compared to CIN 1 or normal cervical tissue 27,31. Interestingly, MMP-2 is constitutively expressed in several cell types, and the accumulation of its active form depends on activation by MT1-MMP, while specific cytokines can operate under MMP-9 transcription regulation 60,61.

Branca et al. 26 observed a strong expression of MT1-MMP in paraffin-embedded invasive cervical cancer samples when compared to normal cervical tissues. This study also showed that all cervical cancer-derived cell lines analyzed (CaSki, C-4II, HT-3, ME-180, MS751, and HeLa) expressed high levels of MT1-MMP and MMP-2. On the other hand, Vazquez-Ortiz et al. 56 observed an upregulation of MMP-10, MMP-11 and MMP-12 and a downregulation of MMP-13 in cervical cancer samples when compared to HSIL samples using cDNA expression arrays. Additionally, it has been observed that keratinocytes expressing E7 from high-risk mucosal and cutaneous HPV types exhibit upregulated expression of MT1-MMP 62.

Moreover, another study analyzed both protein levels and proteolytic activity of 9 MMPs (MMP-1, MMP-2, MMP-3, MMP-7, MMP-8, MMP-9, MMP-13, MMP-14, and MMP-15) and TIMP-1, TIMP-2, and TIMP-3 in normal cervical tissue and LSIL, HSIL and cervical cancer clinical samples using IHC assays and gelatin zymography. The authors observed an increase in the protein levels of MMP-2 and MMP-9 in 13%, 80% and 90% of LSIL, HSIL and cervical cancer samples, respectively 22. MMP-9 specific activity and/or MMP-2 active protein levels were (I) increased in HSIL and cervical cancers when compared to normal control tissues (II) and correlated with increased lymph node metastasis and cancer relapse 22,63. Finally, patients with early stages of cervical cancer showed higher MMP-9 expression and experienced a decreased recurrence-free survival after standard treatment 64.

### Other ECM molecules altered in HPV-associated cancer

The differential expression of specific modulators of ECM protease activity plays a central role in tumor microenvironment remodeling. These positive and negative modulators present an intricate interplay that impacts both their expression and function in HPV-associated tumors 27,31,53,54.

Indeed, the protein levels of a disintegrin and metalloproteinase 17 (ADAM17), amphiregulin (AREG), ECM metalloproteinase inducer (EMMPRIN), and MMP were increased in cervical carcinoma clinical samples when compared with normal adjacent cervical tissues. Moreover, strong ADAM17, AREG, and EMMPRIN protein expression was associated with several poor prognosis parameters, including the presence of lymph node metastasis and advanced tumor stage 65. One of the mechanisms underlying the downregulation of TIMPs in cervical cancer might be through the function of EMMPRIN. Xu et al. 66 showed that exogenous expression of EMMPRIN downregulated TIMP-1 protein in HPV-positive cervical cancer-derived cell lines. These authors have also analyzed EMMPRIN downregulation using iRNA and observed an increase in TIMP-1 protein levels in the same cell lines.

Different types of collagens, including collagen types I, II, III, V, and IX may show increased deposition in the tumor microenvironment 42. Decreased expression or lack of collagen IV is an independent predictive factor for cervical lymph node invasion in patients with early stage cervical carcinoma 67,68. Moreover, increased protein levels of collagen XVII have been reported in cervical cancer clinical samples and were associated with increased local tumor spread. Additionally, this study showed that the *COL17A1* gene was hypomethylated in cervical cancer clinical samples 69.

Syndecan 1, a transmembrane heparan sulfate cell surface proteoglycan that is highly expressed in the normal cervical epithelium (except for the basal cell layer), presents a marked downregulation in invasive cervical carcinomas when compared with CIN samples 36,70,71. Similar to what has been shown for collagen IV, Numa et al. 70 showed that decreased or absent syndecan 1 expression presents an inverse correlation with cervical lymph node invasion but not with overall prognosis in cervical cancer patients. In opposition to these results, it was reported that strong syndecan 1 cytoplasmic expression (without alterations in gene copy number) correlates with improved survival and represents an independent prognostic factor for patients diagnosed with cervical carcinoma 72.

CLDNs and occludins are families of proteins associated with the establishment of tight junctions and epithelial cell polarity and intercellular permeability. Expression levels of CLDN type 1, 2, 4 and 7 proteins were upregulated in HSIL lesions and in invasive cervical tumors when compared with normal cervical tissues 36,73. On the other hand, occludin is expressed in the basal cell layer of normal cervical tissues, and its protein level is decreased in invasive cervical carcinomas when compared with CIN samples. This study indicated that alterations in cell adhesion and ECM structure are a common early feature in cervical cancer progression 73.

The expression of the 67-kd high-affinity laminin�binding protein (67LR) was increased in both CIN and cervical cancer samples when compared with normal cervical tissues 26,74. However, the latter study did not observe a significant capability 67LR to predict either (I) high-risk HPV infection clearance in CIN after treatment or (II) survival in cervical cancer patients 26.

Versican, an ECM proteoglycan, was evaluated in cervical cancer samples by IHC and *in situ* hybridization. The expression of high levels of versican in tumor stromal myofibroblasts was associated with (I) a lower frequency of tumor-infiltrating CD8-positive T cells, (II) increased tumor parametrial invasion and infiltration depth (III) and no change in cervical cancer survival 75. Interestingly, the beta-galactoside-binding protein galectin-1 was more expressed in tumor-adjacent stromal cells when compared with normal cervical tissue-associated stroma 76. Moreover, a higher expression level of galectin-1 was associated with increased local tumor relapse and poor cancer-specific survival in patients with stage I-II cervical tumors after radiation treatment, although it could not predict distant metastasis 77.

In 2015, a new study presented more results on ECM changes in cervical cancer clinical samples when compared with normal cervical tissues obtained from hysterectomy 78. All samples were previously verified for the presence of HPV DNA by nested PCR 79. Formalin-fixed cervical tissues were prepared for tissue microarray (TMA) and analyzed through IHC for 25 different ECM molecules, including laminin-1 and laminin-5, α-smooth muscle actin (αSMA) and fibronectin. Laminin-1 (3.8-fold) and αSMA (5.2-fold) proteins presented increased expression, mainly in cervical tumor-surrounding stroma when compared with normal cervical stroma. Moreover, tumor cells and both tumor and normal tissue-associated fibroblasts were collected from fresh cervical tissue and cultured in order to analyze the secretion of ECM elements. In summary, (I) tumor cells mainly expressed integrin α6β4 laminin receptors and (II) tumor-associated fibroblasts showed higher levels of laminin-α1 and laminin-β1 and lower levels of laminin-5, fibronectin, collagen III, TIMP-1 and the hyaluronan (HA) receptor CD44 when compared with normal fibroblasts 78. Finally, it has been shown that MMP-7 and MMP-9 expression correlate with CD44 expression in skin cancer cells 80,81.

The data discussed above show that alterations in the ECM composition and function are common in HPV-associated lesions and cancers. Altogether, these alterations highlight the complex molecular pathways that lead from initial infection to disease. For instance, the analysis of the impact of HPV on components of the MMP family that have been addressed by studies from different groups around the world have produced a plethora of data that could be used for disease diagnosis and for the identification of targets for therapy. The data summarized here also show that in regard to the mechanisms by which HPV modulates MMP expression and activity, there is still much to learn. Finally, ECM alterations may impact the HPV-infected tissue microenvironment, affecting the recruitment of inflammatory infiltrates, altering the fate of different cell populations present in the tumor and, ultimately, determining the disease progression and prognosis. Therefore, further studies are necessary to understand how HPV proteins affect the dynamic ECM balance in associated pathologies. This will help us to better understand the genesis of the disease and define more suitable clinical interventions. Importantly, a great majority of the ECM alterations described in this review have also been observed in HPV-independent tumors. Therefore, understanding the virus-mediated molecular events leading to ECM disturbance may prove of value for understanding the basic mechanisms of carcinogenesis and the development of more general antitumor approaches.

## AUTHOR CONTRIBUTIONS

Herbster S prepared the text describing ECM alterations in HPV-associated tumors. Paladino A prepared the text describing the general characteristics of HPV and the effect of the virus on ECM alterations present in precursor lesions and prepared the illustration in Figure 1. Freitas S assisted in the preparation of text describing the general characteristics of HPV and the effect of the virus on ECM alterations present in precursor lesions. Boccardo E prepared, revised and corrected all the text.
